# Efficacy and safety of incretin-based therapies in patients with nonalcoholic fatty liver disease: A protocol for a systematic review and meta-analysis

**DOI:** 10.1097/MD.0000000000020695

**Published:** 2020-07-02

**Authors:** Si-min Fan, Xiao-yan Shi, Yan-ping Fan, Lin-lin Yang, Jia Yao, Pei-min Feng

**Affiliations:** a Hospital of Chengdu University of Traditional Chinese Medicine, Chengdu, Sichuan Province; b School of Medicine, Xi’an Jiaotong University, Xi’an, Shanxi Province, P.R. China.

**Keywords:** incretin-based therapies, nonalcoholic fatty liver disease, protocol, systematic review

## Abstract

**Background:**

Nonalcoholic fatty liver disease (NAFLD) is seriously affecting the general health due to its high prevalence and associated risk of liver-related consequences and extrahepatic chronic complications. New drugs are urgently needed for the treatment of NAFLD. The purpose of this meta-analysis is to assess the efficacy of incretin-based therapies in patients with NAFLD.

**Methods:**

We will search 4 databases for relative studies: PubMed, Cochrane Library, Embase, and Web of Science databases and identified all reports of randomized controlled trials (RCTs) published from inception to July 2020. Two authors will independently scan the searched articles, extract the data from included articles, and assess the risk of bias by Cochrane tool of risk of bias. Disagreements will be resolved by discussion among authors. All analysis will be performed based on the Cochrane Handbook for Systematic Reviews of Interventions. Fixed-effects model or random-effects model will be used to calculate pooled estimates of weighted mean difference (WMD) with 95% confidence intervals (CIs) or odds ratio (OR) with 95% CIs.

**Results:**

This systematic review aims to examine the effect of incretin-based therapies on liver histology, liver fat content, liver enzymes, and adverse events in patients with NAFLD.

**Conclusions:**

These findings will provide guidance to clinicians and patients on the use of incretin-based therapies for NAFLD.

## 1 Introduction

Nonalcoholic fatty liver disease (NAFLD) is a clinicopathological syndrome characterized by excessive fat accumulation (>5% hepatic steatosis) in hepatocytes, except secondary causes such as significant alcohol consumption, long-term use of a steatogenic medication, or monogenic hereditary disorders.^[1]^ The great prevalence of NAFLD overall global is around 6% to 35% (median 20%).^[2]^ Even its prevalence is thought to be rising as the unhealthy lifestyle and diet.^[3]^ A large proportion of NAFLD can progress to nonalcoholic steatohepatitis (NASH), which is defined as the presence of > 5% hepatic steatosis and hepatocyte inflammation, with or without fibrosis. In addition, approximately 9% to 20% of NASH patients will progress to cirrhosis.^[4]^ NAFLD is the hepatic manifestation of metabolic syndrome (MS). It is closely related to obesity and MS. The incidence of obesity and MS among NAFLD is estimated to be 51.34% and 42.54%, respectively.^[5–7]^

The current treatment of NAFLD including weight loss and metabolic improvement by lifestyle intervention. A weight loss of ≥7% is associated with histological improvement.^[8]^ Vitamin E is recommended for patients with biopsy-proven NASH without diabetes,^[1]^ but did not have similar efficacy in patients with type 2 diabetes mellitus (T2DM) according to a recent proof-of-concept randomized placebo-controlled trial (RCT).^[9]^ Regarding antidiabetic agents, pioglitazone is recommended for patients with or without T2DM and biopsy-proven NASH.^[8]^ However, it is urgent to identify the best pharmacological approach to treat NAFLD.

Incretin-based therapies, as a new type of hypoglycemic drug, have attracted much attention. It can be divided into 2 categories: glucagon-like peptide-1 receptor agonists (GLP-1Ras) and dipeptidyl peptidase-4 inhibitor (DPP-IVi). In animal models of NAFLD, both GLP-1Ras^[10]^ and DPP-IVi^[11]^ improve hepatic steatosis and its related pathways. In open-label prospective trials, sitagliptin has been found to improve liver histology,^[12]^ plasma aminotransferases,^[13]^ intrahepatic lipid.^[14]^ Liraglutide also has been positive with an improvement in hepatic steatosis ^[15–17]^ or liver histology.^[18]^ However, in some clinical trials, negative studies have been also reported with both sitagliptin^[19,20]^ and liraglutide.^[21]^ The results are not consistent. The objective of this meta-analysis is to explore the efficacy of Incretin-based therapies in patients with NAFLD.

## 2 Methods

### 2.1 Registration

Our meta-analysis protocol was registered in the International platform of registered systematic review and meta-analysis protocols (INPLASY) as number INPLASY202050045.

This study will be designed in accordance with the Preferred Reporting Items for Systematic Reviews and Meta-analysis guidelines.^[22]^

### 2.2 Eligibility criteria

The criteria for study inclusion will be: study design: RCTs with any follow-up duration and sample size will be allowed; population: adult (age ≥ 18 year) patients with a definitive diagnosis of NAFLD or NASH by histologic or imaging evidence (ultrasound, computer tomography, or magnetic resonance imaging; intervention: GLP-1RAs (liraglutide, exenatide, albiglutide, lixisenatide, and dulaglutide) or DPP-IVi (sitagliptin, vildagliptin, saxagliptin, alogliptin, linagliptin, gemigliptin, and teneligliptin) at any dose and route; control: placebo or other active agents; outcomes: primary outcomes are liver histology including steatosis score, hepatocellular ballooning score, and lobular inflammation score and liver fat content. Secondary outcomes are liver enzymes including alanine aminotransferase (ALT), aspartate transaminase (AST), and γ-glutamyl transferase (GGT) and adverse events.

The exclusion criteria will be as follows: not RCTs (e.g., animal, in vitro study, observational studies, etc); studies not assessing primary data; letter to the editor, conference papers, and articles only available in abstract form.

### 2.3 Search methods for the identification of studies

Two reviewers (SF and XS) will independently search English-language publications on PubMed, Embase, Cochrane Library, and Web of Science databases from inception to July 2020. According to the PICOS principle, the main key search words will be: “exenatide” OR “liraglutide” OR “albiglutide” OR “lixisenatide” OR “dulaglutide” OR “glucagon-like peptide 1” OR “sitagliptin” OR “vildagliptin” OR “saxagliptin” OR “alogliptin” OR “linagliptin” OR “gemigliptin” OR “teneligliptin” OR “dipeptidyl peptidase-4 inhibitor” AND “nonalcoholic fatty liver disease” OR “NAFLD” OR “nonalcoholic fatty liver∗” OR “nonalcoholic steatohepatiti∗” OR “NASH” OR “fatty liver∗.” The reference lists of reviewed articles will be manually searched for additional relevant studies and the ClinicalTrials.gov registry will also be searched for unpublished trials. When incomplete information is available, attempts will be made to contact the study investigators for additional information. A third reviewer (YF) will be involved in a discussion for any disagreements.

### 2.4 Data collection

#### 2.4.1 Study selection

**Figure 1:**
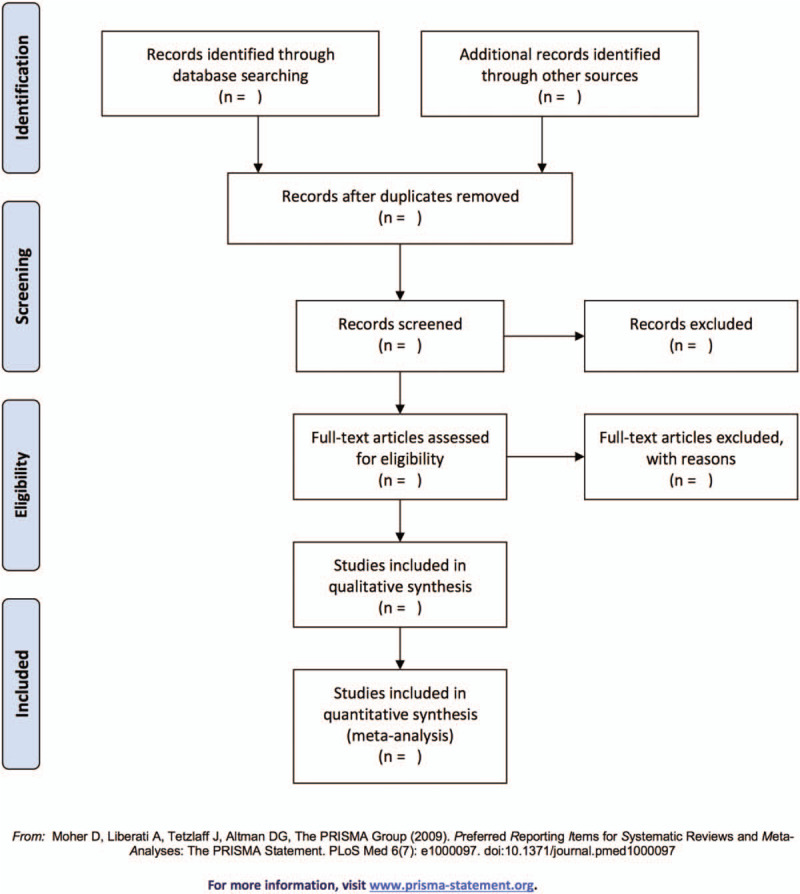
Flow diagram of study selection.

Two reviewers (SF and XS) will independently review all identified data based upon the exclusion and inclusion criteria and remove duplicate literature. Titles, abstracts, and full-text articles will be screened and data will be extracted independently by those reviewers, with discrepancies discussed with the third reviewer (YF). The selection process will be shown in a Preferred Reporting Items for Systematic Review and Meta-analysis flow chart (Fig. 1).

#### 2.4.2 Data extraction

Using a predefined data extraction sheet, two reviewers (SF and XS) will independently extract data for review. A third reviewer (YF) will be involved in a discussion for any disagreements. The following information will be extracted from the included studies: the first author, published year, study location, study design, inclusion/exclusion criteria, sample size, participants’ baseline characteristics, intervention characteristics, control, and outcome data.

### 2.5 Quality assessment

Based on the Cochrane Handbook for Systematic Reviews (version 5.3.0), we will assess the methodological quality of all studies.^[23]^ The risks of bias will be classified as low, unclear, or high by evaluating the 7 components as random sequence generation, allocation concealment, blinding of outcome assessment, blinding of participants and personnel, incomplete outcome data, selective outcome reporting, and other bias. Two independent reviewers (SF and XS) will conduct this assessment, and a third reviewer (YF) will be consulted for any disagreements.

### 2.6 Data analysis

#### 2.6.1 Measurement of the treatment effect

All statistical analysis will be performed using STATA 15.0 software. We will calculate odds ratio (OR) and 95% confidence intervals (CIs) of outcomes when it is a dichotomous variable. Outcomes will be presented as the weighted mean difference (WMD) and 95%CIs when it is a continuous variable. If raw data is not reported, we will impute unreported means ± SDs using established methods via other information (e.g., CIs or median and interquartile range, etc) provided in the publication.^[24]^ If only the pre and post intervention period data are provided, we will calculate the change of mean ± SDs using the formula recommended in the Cochrane Handbook Version 5.3.0.^[23]^

#### 2.6.2 Dealing with missing data

If raw data is not directly provided in the text or tables, figures in the study will be consulted. Once relevant details are insufficiently reported in studies, the authors will be contacted and the ClinicalTrials.gov register will be searched for further information. If unsuccessful, the missing data will be calculated from the raw numbers and reported *P* values.^[23,24]^

#### 2.6.3 Assessment of heterogeneity

Statistical heterogeneity will be assessed by the Cochran Q test and I^2^ statistic (*P* value < .10 or I^2^ statistic > 50% will be defined as substantial heterogeneity). A random-effects model will be applied in the presence of heterogeneity. In other cases, the fixed-effects model will be employed.

#### 2.6.4 Assessment of reporting biases

Using the funnel plot if more than 10 studies are included. Considering the subjectivity of funnel chart, we will also use Egger and Begg tests to evaluate publication bias, *P* < .05 will be considered statistically significant.

#### 2.6.5 Subgroups analysis and sensitivity analysis

Subgroup analysis is prespecified according to intervention drugs (GLP-1Ras or DPP-IVi). Sensitivity analysis will be performed by removing a single trial each time and repeating the meta-analysis to assess the reliability and stability of the pooled results.

## 3 Discussion

NAFLD is seriously affecting the general health due to its liver-related consequences (liver cirrhosis and hepatocellular carcinoma) and its great risk for extra-hepatic chronic complications (T2DM, MS, and cardiovascular disease).^[1]^ The role of incretin-based therapies as a potential treatment for NAFLD has attracted much attention. Numerous studies have demonstrated the improvement effect of incretin-based therapies on liver histology,^[12]^ intrahepatic lipid,^[14]^ and liver enzymes.^[13]^ However, negative studies have been also reported in some clinical trials.^[19,20]^ The conclusion is still controversial. We will systematically review RCTs to evaluate the efficacy and safety of incretin-based therapies in NAFLD patients. These findings will provide guidance to clinicians and patients on the use of incretin-based therapies for T2MD with NAFLD.

## Author contributions

**Conceptualization:** Si-min Fan, Pei-min Feng.

**Data analysis:** Si-min Fan, Lin-lin Yang.

**Data extraction:** Xiao-yan Shi, Si-min Fan, Yan-ping Fan.

**Funding acquisition:** Pei-min Feng.

**Methodology:** Pei-min Feng.

**Project administration:** Pei-min Feng.

**Resources:** Pei-min Feng.

**Software:** Lin-lin Yang, Jia Yao.

**Writing – original draft:** Si-min Fan, Xiao-yan Shi.

**Writing – review & editing:** Si-min Fan, Yan-ping Fan.
